# *Staphylococcus epidermidis* SAS1: new probiotic candidate for obesity and allergy treatment their mechanistic insights and cytotoxicity evaluation

**DOI:** 10.3389/fmicb.2025.1546687

**Published:** 2025-04-30

**Authors:** Sonia Sharma, Aarjoo Sharma, Gurleen Kaur Sodhi, Nancy George, Khaloud Mohammed Alarjani, Arkadeep Mukherjee, Santosh Kumar Rath, Ramandeep Kaur, Vagish Dwibedi

**Affiliations:** ^1^University Institute of Biotechnology, Chandigarh University, Mohali, India; ^2^Department of Microbiology, Panjab University, Chandigarh, India; ^3^Department of Botany and Microbiology, College of Science, King Saud University, Riyadh, Saudi Arabia; ^4^Department of Civil Engineering, Yeungnam University, Gyeongsan, Republic of Korea; ^5^School of Pharmaceuticals and Population Health Informatics, Faculty of Pharmacy, DIT University, Dehradun, India; ^6^Department of Biotechnology, Chandigarh Group of Colleges, Landran, India

**Keywords:** probiotic candidate, *Staphylococcus epidermidis*, obesity, allergy, therapeutic treatment

## Abstract

**Background:**

Probiotics are live bacteria that provides numerous healthy and beneficial effects to the consumers. The present study aimed to investigate the effect of a probiotic candidate *Staphylococcus epidermidis* SAS1, in immunoregulation and obesity management.

**Methods:**

: This probiotic candidate was isolated from a soil sample collected from a region of fruit waste decomposition. *In vitro* cytotoxicity was assessed using the THP-1 (human leukemia monocytic cell line) cells using MTT assay.

**Results:**

An IC50 value of 47.52 ± 0.18 μg/mL and cell shrinkage were observed along with the release of cellular content of THP-1 cells. The higher production of reactive oxygen species and lesser release of interleukins (IL-4, 5, and 13) are attributed to the antiallergic potential of this strain. Furthermore, in vitro cytotoxicity evaluation using 3T3-L1 cells identified this strain as a promising candidate for anti-obesity treatment. The observed IC50 value was 514.4 ± 0.061 μg/mL.

**Discussion:**

This extract was shown to have good lipase-inhibiting enzyme activity and was reported to prevent adipogenesis, depicted by increased HDL levels and decreased LDL and triglyceride levels. These results suggested that Staphylococcus epidermidis SAS1 may have therapeutic use in the treatment of obesity and allergies.

## 1 Introduction

The gut microbiota is regarded as a “vital organ” of the body due to its extensive interactions with various organs through endocrine, neural, humoral, and metabolic pathways. The composition of the gut microbiota is significantly shaped by numerous host factors, such as diet, environmental conditions, physical activity, lifestyle choices, and age. The acknowledgment of the relationship between human health and the gastrointestinal system dates back to 400 B.C., when Hippocrates remarked that “death sits in the bowls” (AboNahas et al., 2022). Changes in the gut microbiome impact the gastrointestinal system and other organs. Numerous human health conditions, such as bowel syndromes, autoimmune disorders, diabetes, hypertension, obesity, and cardiovascular issues, have been associated with alterations in gut microbiota (Afzaal et al., 2022; Vijay and Valdes, 2022).

There are undoubtedly many drugs accessible on the market; however, they can also have a variety of detrimental effects on the human body. Consequently, it is essential to maintain a balanced gut microbial population to ensure a harmonious coexistence between the microbiome and the host. Probiotics can potentially enhance or avert intestinal and systemic diseases by restoring the balance of the gut microbiome and providing beneficial functions to gut microbial populations. The World Health Organization (WHO) states that probiotics are live microbes that provide health benefits to the human body when consumed in large quantities (Sionek et al., 2023) and are ‘Generally Regarded as Safe (GRAS)’ for consumption (Růžičková and Kohout, 2023).

Probiotic research has shown significant success in treating various diseases, such as obesity, hypertension, stress, and gastrointestinal disorders (Vlachou et al., 2020; Wiciński et al., 2020; Harsh and Sangita, 2022). Obesity is a metabolic condition that affects health and results from excess white adipose tissue. These conditions have become a global problem in today’s society, increasingly affecting people of all ages due to poor diet and lifestyle choices. Alarmingly, it is linked to many health problems, such as diabetes, cancer, heart disease, osteoarthritis, and high blood pressure. Obesity is when the body stores too much fat, leading to changes in body mass index (BMI) (Salmón-Gómez et al., 2023).

Overweight is described by a body mass index (BMI) greater than or equal to 25 kg/ m^2^. It is estimated that by 2022, approximately 2.5 billion adults worldwide will be classified as overweight, and 890 million people will be obese. In addition, a total of 37 million children and 390 million adolescents aged 5 and older are classified as overweight (Okunogbe et al., 2022). It is estimated that by 2030, approximately 1.12 billion adults, approximately 20% of the world’s population, will be classified as obese. Despite the availability of many medications, obesity, and its effective management are still difficult because these treatments may cause adverse side effects or require adverse interventions such as physical exercise, dietary changes, and weight loss strategies (Fruh, 2017). Therefore, developing or identifying safe and reliable probiotics is necessary.

Allergy is characterized by an abnormal immune system in which the body responds immediately to various lifestyle, genetic, and environmental factors (Sidhu and Nehra, 2021). Allergic diseases have increased in recent years, especially in developing countries. As this trend continues, the WHO has listed it as “a health problem of international concern” (Shen et al., 2019). By 2050, 4 billion people worldwide are expected to suffer from allergic symptoms, posing a major crisis for public health and society in general. Allergens attack our lymphocytes, causing the production of IgE antibodies and the release of bioactive mediators from mast cells and basophils. Therefore, allergy symptoms such as asthma, urticaria, allergic rhinitis, and atopic eczema may occur. However, a complete cure is still possible.

This study evaluated the novel probiotic *Staphylococcus epidermidis* SAS1, identified under GenBank accession number PP 177331.1, for its ability to prevent obesity and allergies. *Staphylococcus epidermidis*, a bacterium found in the skin, has been shown to have many health benefits, such as strengthening the immune system, acting against microbial invasion, reducing the damage to the body, and reducing pain after injury (Keshari et al., 2019;. Menconi et al., 2014). A review of the available literature shows that *Staphylococcus epidermidis* has not been studied for its ability to prevent obesity and allergies. This study aimed to evaluate the cytotoxic effects of THP1 cells to investigate the production of reactive oxygen species (ROS) and to measure the expression levels of various interleukins, especially IL-4, IL-5, and IL-13. Anti-obesity properties were also evaluated by lipase inhibition test and measurement of triglyceride and glycerol levels.

## 2 Materials and methods

### 2.1 Material

Inhibitor (Orlistat, Cayman Chemicals-Cas no 96829-58-2), Enzyme (Lipase, SRL chem, Cat no 60770), p-nitrophenyl palmitate- Merk (Sigma), Cat no N2752-1G, Human IL-4 GENLISATM ELISA- Cat No. KB1066 kit, Human IL-5 GENLISATM ELISA- Cat No. KB1067 kit, Human IL-13 GENLISATM ELISA- Cat No. KB1076 kit. Triglyceride assay kit (ClinReact Sypa kit), Phosphate-buffered saline (PBS).

### 2.2 Probiotic candidate

The *Staphylococcus epidermidis* SAS1 strain (Genbank accession number PP177331.1) was obtained from a soil sample collected in the fruit waste area of the Grain Market in Chandigarh, India (30.7233°N, 76.8080° E).

SAS1 was biochemically characterized following the procedure outlined in Bergey’s Manual of Systematic Bacteriology. This strain was maintained on nutrient agar (NA) plates and stored at −20°C for future studies (Bergey, 1994).

### 2.3 Production and extraction of bioactive metabolites

To produce bioactive metabolites, 1 mL of *S. epidermidis* culture was inoculated into 200 mL of Mueller Hinton broth (MHB) and kept at 37 ± 2°C with shaking at 120 rpm. After the incubation period, the mixture was thoroughly homogenized using a motor and pestle and then transferred into 250 mL centrifuge bottles, which were subsequently stored at −80°C for 20 h. The bottles were then thawed in a water bath set at 65°C for 1 h. Following this, the mixture underwent centrifugation at 15,000 g at 4°C for 40 min. The supernatant was carefully collected and poured into a 1 L glass bottle.

The solvent used in the liquid–liquid extraction process was chloroform. After being mixed in a 3:1 ratio, the solvent and supernatant were shaken vigorously. This process was carried out three times. For 20 to 24 h, the resultant mixture was then kept overnight at 4°C for overnight. The organic layer was carefully transferred into a 50 mL screw-cap tube after the aqueous layer was removed. Centrifuging the mixture for 20 min at 4000 rpm produced the interfacial layer (Cheng et al., 2020). Further, the 20 mL of extracted bioactive metabolites was used to assess its potential for anti-allergic and anti-obesity effects.

### 2.4 Identification of bioactive metabolites using LC–MS/MS analysis

Bioactive metabolites were analyzed using LC–MS/MS on a Waters, SYNAPT-XS HDMS, UK, with UPLC ACQUITY H CLASS Series System of model DBA064 (Waters Corporation, UK). The separation was carried out using C18 Waters, Acquity BEH 2.1*100 mm 1.7um. Throughout the analysis, the sample temperature was held at 15°C and the column was kept at 40°C. The desolvation temperature was at 550°C, and the desolvation gas Flow was kept at 950Lts/Hr. The main working parameters for MS were: ionization type- ESI Positive acquisition mode-MRM, run time 45 min, mass range (m/z) 50–1,200 m/z, collision energy 4 eV (ramp) (for positive mode) and cone voltage 50 V (for positive mode), capillary voltage 3.22 kV (for positive mode), source temperature 120°C, cone gas flow 50 L/h, desolvation gas flow 950 L/h. The elution was carried out in positive mode [ES+] at a flow rate of 0.2 mL/min using gradient mobile phase, 0.1% Formic Acid + LC–MS Grade water (Solvent A) and 0.1% Formic Acid + Acetonitrile (Solvent B). The volume ratio of solvent B was changed as follows, 5% B for 0–1 min, 5–25% B for 1–5 min, 25–35% B for 5–8 min, 35–45% B for 8–11 min, 45–55% B for 11–14 min, 55–90% B for 14–20 min, 90–5% B for 20–20.1 min, and 5% B for 20.1–23 min. 5 μL of the test solution was injected for the phytochemical screening, and the chromatographs were recorded for 23 min (Wang et al., 2023; Sidhu and Nehra, 2021).

### 2.5 Probiotic as anti-allergic candidate

#### 2.5.1 In-vitro cytotoxicity evaluation

The THP-1 (human leukemia monocytic cells) cell line was procured from the National Center for Cell Sciences (NCCS), Pune, India, and maintained in Dulbecco’s Modified Eagle Medium (DMEM). The cell line was cultured in T75 cell culture flasks in a total volume of 20 mL of with DMEM supplemented with 10% FBS, 0.2 mM L-glutamine, and antibiotic solution (1X) containing penicillin (50 μg/mL), streptomycin (50 μg/mL), and neomycin (100 μg/mL). The cell line was kept at 37°C in a humidified 5% CO2 incubator.

The cytotoxic effects on THP-1 cells, were assessed using the 3-[4,5-dimethylthiazol-2-yl]-2,5-diphenyl tetrazolium bromide (MTT) assay. The density of 10,000 cells was plated in each well of a 96-well plate and incubated at 37°C for 24 h. RPMI 1640 medium containing 10% Fetal Bovine Serum (FBS) and 1% antibiotic solution, maintained in an atmosphere of 5% CO_2_.

The cells were treated with various concentrations (ranging from 0 to 1,000 μg/mL) of *S. epidermidis* SAS1 metabolites. Following a 24-h incubation period, 300 μL of MTT solution was added. The incubation continued for an additional 2 h. Subsequently, the culture supernatant was discarded, and the cell layer matrix was mixed in 100 μL of Dimethyl Sulfoxide (DMSO). The absorbance of the samples was measured at 540 nm and 660 nm using an ELISA plate reader (iMark, Biorad, USA). The IC_50_ value was determined utilizing Graph Pad Prism-6 software. Images were captured with an inverted microscope (Olympus ek2) using a Camera (AmScope digital camera 10 MP Aptima CMOS) (Fotakis and Timbrell, 2006).

#### 2.5.2 Reactive oxygen species (ROS) estimation of THP-1 cell line

A density ranging from 5,000 to 10,000 THP-1 cells was transferred into each well of 96-well plates. Each well contained 1 mL of DMEM medium enriched with 10% FBS and 1% antibiotic solution. The seeded medium was maintained in an environment with 5% CO_2_ and kept at 37°C for 24 h. Following substituting the old medium with the new culture medium, the cells underwent an additional incubation period of 24 h. Upon completion of the incubation, the medium was dispose of, and the cells were detached using trypsin–EDTA and subsequently collected in a 1.5 mL tube. The cells were then washed once with 500 μL of chilled PBS. The cell pellet was resuspended in 100 μL of PBS containing 2 μM DCFDA, and the samples were analyzed using a Flow Cytometer (BD FACS Calibur, USA) within 1 h. The acquired data were analyzed using Flowing Software version 2.5.1 (Al-Oqail, 2021).

#### 2.5.3 Protein expression analysis using ELISA for IL-4

The protein expression analysis for IL-4 was conducted utilizing the Human IL-4 GENLISATM ELISA kit (Cat. no. KB1066). A volume of 100 μL of the standard solution (IL-4 at a concentration of 1 μg/mL) along with test sample (SAS1 metabolites) was added to the plate. Subsequently, 50 μL of the diluted biotin-conjugated detection antibody was added to each well.

The plate was securely sealed and incubated for 30 min at a temperature of 37°C. The plate was rinsed four times using wash buffer (1X), and excess buffer was blotted by firmly tapping the plate upside down on absorbent paper. A volume of 100 μL of diluted Streptavidin-HRP solution was added to each well, after which the plate was sealed again and incubated for 30 min at 37°C. The plate underwent another wash with the 1X wash buffer following this incubation. After washing, 100 μL of TMB substrate (3,3′,5,5’-Tetramethylbenzidine) solution was added, and the plate was kept in the dark for 30 min. 100 μL of stop solution was added to each well for the termination of the reaction. Finally, the absorbance was measured at 450 nm after a 10-min interval.

#### 2.5.4 Protein expression analysis with ELISA for IL-5 and IL-13

Protein expression of IL-5 and IL-13 was assessed utilizing the Human IL-5 GENLISATM ELISA kit (Cat. no. KB1067). 100 μL of the standard solutions (conc. of 1 μg/mL) and test sample (SAS1 metabolites) were added to the ELISA plates. Subsequently, 50 μL of the diluted biotin-conjugated detection antibody was added to each well. The plates were then securely sealed and kept at 37°C for 30 min. Following incubation, the plates underwent four washes with wash buffer (1X), and the plate was firmly tapped upside down on absorbent paper to remove any remaining buffer.

Following the addition of 100 μL of diluted Streptavidin-HRP solution to each well, the plates were sealed and kept at 37°C for 30 min. The plates were rinsed with wash buffer (1X) after the incubation. Subsequently, 100 μL of TMB substrate (3,3′,5,5’-Tetramethylbenzidine) solution was added, and the plate was incubated in the dark for 30 min. Upon completion of the incubation period, 100 μL of stop solution was added to each well to terminate the reaction. The absorbance of the plates was measured at 450 nm after the incubation period of 10 min.

### 2.6 Probiotic as anti-obesity candidate

#### 2.6.1 In-vitro cytotoxicity analysis using 3T3 L1 for adipogenesis determination

The 3T3 L1 (mouse adipocyte cell line) obtained from NCCS Pune India, and maintained in Dulbecco’s Modified Eagle Medium (DMEM). The cell line was cultured in T75 cell culture flasks in a total volume of 20 mL of with DMEM supplemented with 10% FBS, 4.0 mM L-glutamine, NaHCO3 (3.7 g/L), glucose (4.5 g/L). The cell line was kept at 37°C in a humidified 5% CO2 incubator.

The cytotoxicity assessment was conducted using the 3T3 L1 by MTT assay for adipogenesis analysis. 10,000 cells were seeded in each well of a 96-well plate using DMEM (Dulbecco’s Modified Eagle Medium) and incubated at 37°C for 24 h. The DMEM was enriched with 10% FBS and 1% antibiotic solution in a humidified incubator with 5% CO_2_. Following the 24-h incubation period, the cells were exposed to varying concentrations (0–1,000 μg/mL) of *S. epidermidis* SAS1 bioactive metabolites and incubated for 24 h. Cells without treatment were referred as Control, and cells without MTT were referred as Blank.

Following a 24-h incubation period, MTT Solution was added to the cell culture and kept for 2 h. Upon completion of the experiment, the culture supernatant was discarded. The cell layer matrix was then dissolved in 100 μL of DMSO, and absorbance was measured at 540 nm and 660 nm using an ELISA plate reader (iMark, Biorad, USA). The IC_50_ value was determined utilizing Graph Pad Prism −6 software. Images were obtained with an inverted microscope (Olympus ek2) and an AmScope digital camera (10 MP Aptima CMOS). The 50% inhibitory concentration (IC_50_) was expressed as Mean ± SEM (Standard Error of Mean) (Morgan, 1998; Tihăuan et al., 2020).


$$\%\mathrm{Viable cells}={\left(A_{\mathrm{test}}/A_{\mathrm{Control}}\right)}^{*}100$$

Here, A_test_, absorbance of test samples; A_Control_, absorbance of control.

#### 2.6.2 Lipase enzyme inhibition assay

Lipase activity was assessed utilizing p-nitrophenyl palmitate as the substrate. A reaction buffer of 4 mL was prepared by combining 3,500 μL of a 1 M Tris-Cl buffer at pH 8 with 500 μL of the substrate. The samples ranging from 0 to 4,000 μg/mL were diluted in the lipase reaction buffer. An enzyme solution of 10 μL, containing 1 mg/mL of lipase, was prepared in the Tris-Cl buffer at pH 8 and subsequently added to a designated well of a 96-well plate. Following this, 10 μL of the samples were added, and the resulting mixture was kept for 10 min.

The untreated reaction mixture served as the control. Following incubation, 80 μL of the substrate was added to each reaction mixture, and the plate was subsequently incubated at room temperature for 5 min. Absorbance was measured at 415 nm utilizing a microplate reader (iMark, BioRad). An inhibitor, orlistat (50 μg/mL), was used as a positive control. The IC_50_ value was determined using Graph Pad Prism 6 software (Kumar et al., 2013).


$$\%\mathrm{Inhibition}=\left(A_{\mathrm{Test}}-A_{\mathrm{Control}}/A_{\mathrm{Control}}\right)\times 100$$

### 2.7 Measurement of glyceride in cell line: HDL and LDL cholesterol

The glyceride content was assessed in 3T3-L1 adipocytes utilizing an MTT assay (ClinReact Sypa kit). Initially, cells were collected from the flask when they reached 90% confluency and subsequently plated in 96-well plates and 50 mm dishes at a density of 10,000 cells per well in DMEM medium. After that, cells were then kept at 37°C for 24 h in an atmosphere with 5% CO_2_. Following this incubation, the DMEM medium was discarded, and fresh culture medium was added to each plate well. A volume of 5–50 μL (representing 10% of the total medium in the cell culture) of the prepared treatment was added to the specified wells, and the treated plates were incubated for an additional 24 h.

For the MTT assay, two sets of 96-well plates were utilized, one designated for measuring HDL cholesterol and the other for LDL cholesterol. In each well, 150 μL of HDL R1 or LDL R1 was added for both the calibrator and test samples. Subsequently, 10 μL of the calibrator and 10 μL of the sample were added into their corresponding wells. The mixtures were thoroughly combined and incubated for 5 min at 37°C. After this initial incubation, 50 μL of HDL R2 or LDL R2 was added to each well. Following another mixing step, the absorbance (A1) was recorded after an additional 5-min incubation at 37°C. Finally, the absorbance (A2) was measured at a wavelength of 595 nm. Cells that were not treated served as the control group (Rifai et al., 2000).


$$\mathrm{Concentration}=A2_{\mathrm{test}}–A1_{\mathrm{test}}/A2_{Cal}–A1_{Cal}\times Cal.{\mathrm{Concentration}}^{*}$$

Here, A1, First Absorbances before 2nd incubation; A2, Final Absorbances After 2nd incubation; Cal. Concentration* = Calibrator Concentration (55 mg/dL for HDL and 120 mg/dL for LDL).

### 2.8 Triglyceride measurement

Initially, 3T3-L1 adipocyte cells were collected when they reached 90% confluency and subsequently plated in 96-well plates and 50 mm dishes at a density of 10,000 cells per well using DMEM medium. The cells were then incubated for 24 h at 37°C in an atmosphere containing 5% CO_2_. After this incubation, the medium was discarded, and fresh culture medium was introduced into each plate well. A volume of 5–50 μL, representing 10% of the total medium in the cell culture, was added to the specified wells, and the treated plates were incubated for an additional 24 h.

Cleaned plates were designated and labeled as calibrators and tests (control and sample). The measurement process was conducted following the ClinReact Sypa kit protocol. In each well, 1,000 μL of Reagent was added. Subsequently, 10 μL of the calibrator and 10 μL of the sample were added into their corresponding wells. The mixtures were thoroughly agitated and incubated for 5 min at a temperature of 37°C. Finally, the absorbance was taken at a wavelength of 490 nm. Untreated cells were considered as Control group (Grossman et al., 1976).


Triglyceridemg/dL=Absorbance of Sample×K−Factor

K- Factor = Calibrator or Standard Concentration (mg/dL) / Calibrator or Standard Absorbance.

### 2.9 Statistical analysis

All the experiments were performed in triplicate, and the data was expressed as mean ± standard deviation and analyzed by Tukey’s *Post hoc* analysis.

## 3 Results

### 3.1 Probiotic candidate

In this study, a novel probiotic *Staphylococcus epidermidis* SAS1, having accession number PP177331.1 is determined for its anti-obesity and anti-allergic potential. Biochemical testing of SAS1 has been done before. Moreover, SAS1 is a non –pathogenic strain which was conferred by observing negative observation of hemolysis, Dnase, gelatinase and urease tests (Supplementary Table S1).

### 3.2 Identification of bioactive metabolites using LC–MS/MS analysis

Using UPLC ACQUITY H Waters LC–MS with a comprehensive tally of 75 bioactive compounds was identified in the positive mode of ionization (Supplementary Table S2). Data from precise mass measurements of the targeted analytes and information gleaned from mass fragmentation patterns were crucial for identifying compounds. Additional verification was accomplished by utilizing spectral libraries, including Chemspider, KEGG, METLIN search, Progenesis Metascope, NPAtlas, and PlantCyc. The identification of few major compounds 1-oleoyl-2-palmitoyl-sn-glycerol, 6α-Hydroxytestosterone, 16β-hydroxytestosterone, 3-Pyridinecarboxaldehyde, Undecanol, 2-Acetylaminofluorene, Cyclo(L-Trp L-Pro), N-Methylcytidine, 9-Hydroxy-10-methoxy-3,12-didehydrogalanthan-1-one, S-[(1Z)-N-Hydroxy-6-(methylsulfanyl)hexanimidoyl]cysteine, L-(+)-Penicillamine, Cinchonidinone, S-(1H-Indol-3-ylmethyl)-L-cysteine, Ancymidol, (2R,2’R)-2,2′,3,3’-Tetrahydroxy-beta,beta-caroten-4-one, Cinchoninone, Prosolanapyrone II, 7,8-Diaminononanoic acid, beta-D-Fructofuranosyl 4-O-(3-methylbutanoyl)-alpha-D-glucopyranoside, Lipoic acid, L-Methionine Sulfoximine, 5-Methoxy-6-methyl-1H-benzimidazole, 3,6-Nonadienal, 7-(3-methylbut-2-enyl)-L-tryptophan, 1D-3-amino-1-guanidino-1,3-dideoxy-scyllo-inositol 6-phosphate, L-(−)-Camphor, Crinine, Oxynicotinamide, (5E,7E,10R,12Z,14E,17R,18S,22S)-22-Ethyl-10,18-dihydroxy-17-methyloxacyclodocosa-3,5,7,12,14-pentaene-2,16-dione, (−)-beta-phellandrene, (2S,3R,5S)-3-(2-Aminoethyl)-7-oxo-4-oxa-1-azabicyclo[3.2.0]heptane-2-carboxylic acid, Porphobilinogen, 7-(3-methylbut-2-enyl)-L-tryptophan, Cyclopeptine, (5Z)-5-[(1,6-Dihydroxy-2,4-cyclohexadien-1-yl)imino]-5-hydroxy-L-norvaline, Proclavaminic acid, (Z,5Z)-5-{[(1Z,2S)-6-Amino-1-{[(1Z,2R)-1-{[(1R)-1-carboxyethyl]imino}-1-hydroxy-2-propanyl]imino}-1-hydroxy-2-hexanyl]imino}-N-[(2S)-2-amino-1-hydroxypropylidene]-5-hydroxy-L-norvaline, 2,4-diacetamido-2,4,6-trideoxy-beta-L-altrose,(3beta,12beta,16beta,21beta)-16,21,23-Trihydroxy-30-oxo-12,13-epoxyoleanan-3-ylbeta-D-glucopyranosyl-(1- > 2)-[beta-D glucopyranosyl-(1- > 4)]-alpha-L-arabinopyranoside, (Z)-N-[(2S)-2-{(Z)-[(2S,3S,6R,7S)-2,3-Diamino-1,6,7-trihydroxy-8-{1-[hydroxy(imino)methyl]-2 imino4imidazolidinyl} octylidene] amino}-1-hydroxypropylidene]-L-valine were confirmed through mass fragmentation patterns. Our research especially focused on identifying major phytochemicals using UPLC ACQUITY analysis that resulted in the identification of 75 compounds through the positive mode of ionization techniques (Supplementary Table S2).

### 3.3 Anti-allergic potential of probiotic *Staphylococcus epidermidis* SAS1

#### 3.3.1 In-vitro cytotoxicity evaluation of the compound- THP-1

**Figure 1:**
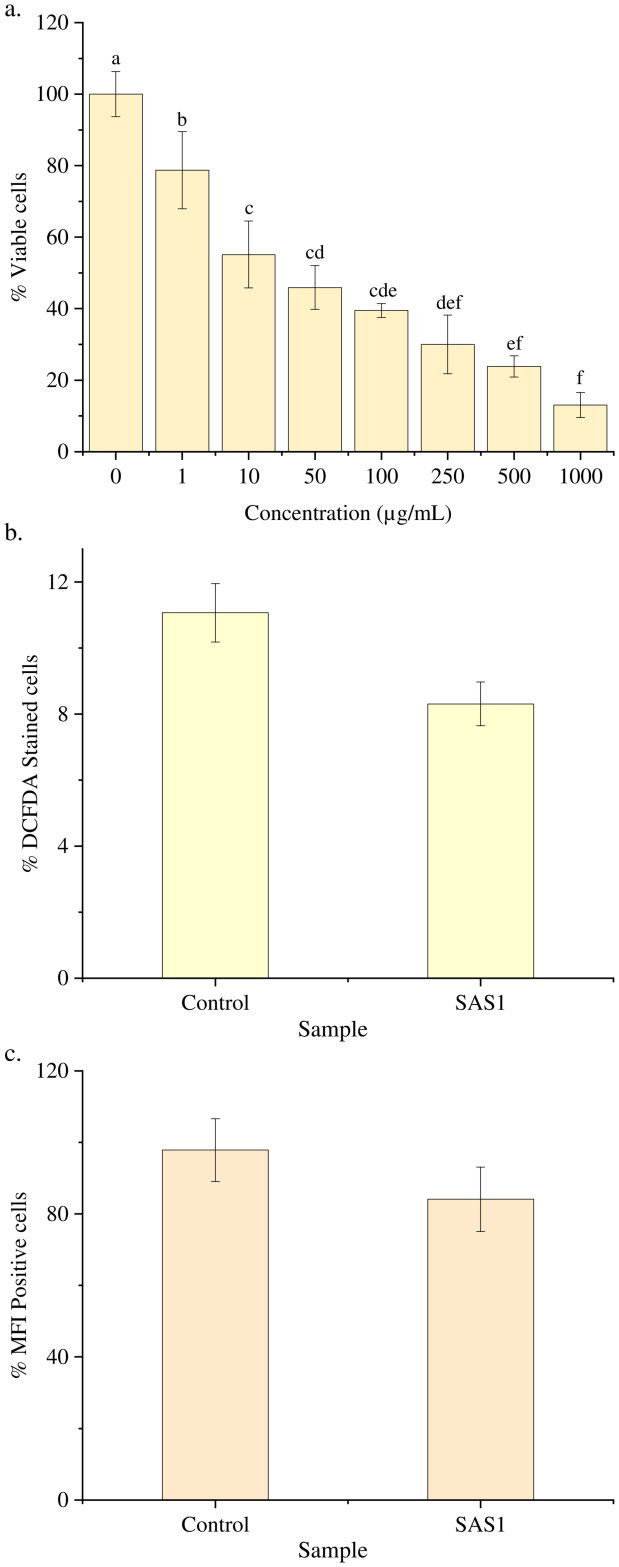
**(a)** Reduction in the number of THP-1 cell line on increasing the exposure of *S. epidermidis* SAS1 bioactive metabolites; **(b)** Flow cytometry % DCFDA stained cells; **(c)** Medium fluorescence intensity (MFI) of reactive oxygen species (ROS). The data represents mean *n* = 3 ± SD. Mean with different superscript letters are different by Tukey’s *post hoc* test (*p* < 0.05).

**Figure 2:**
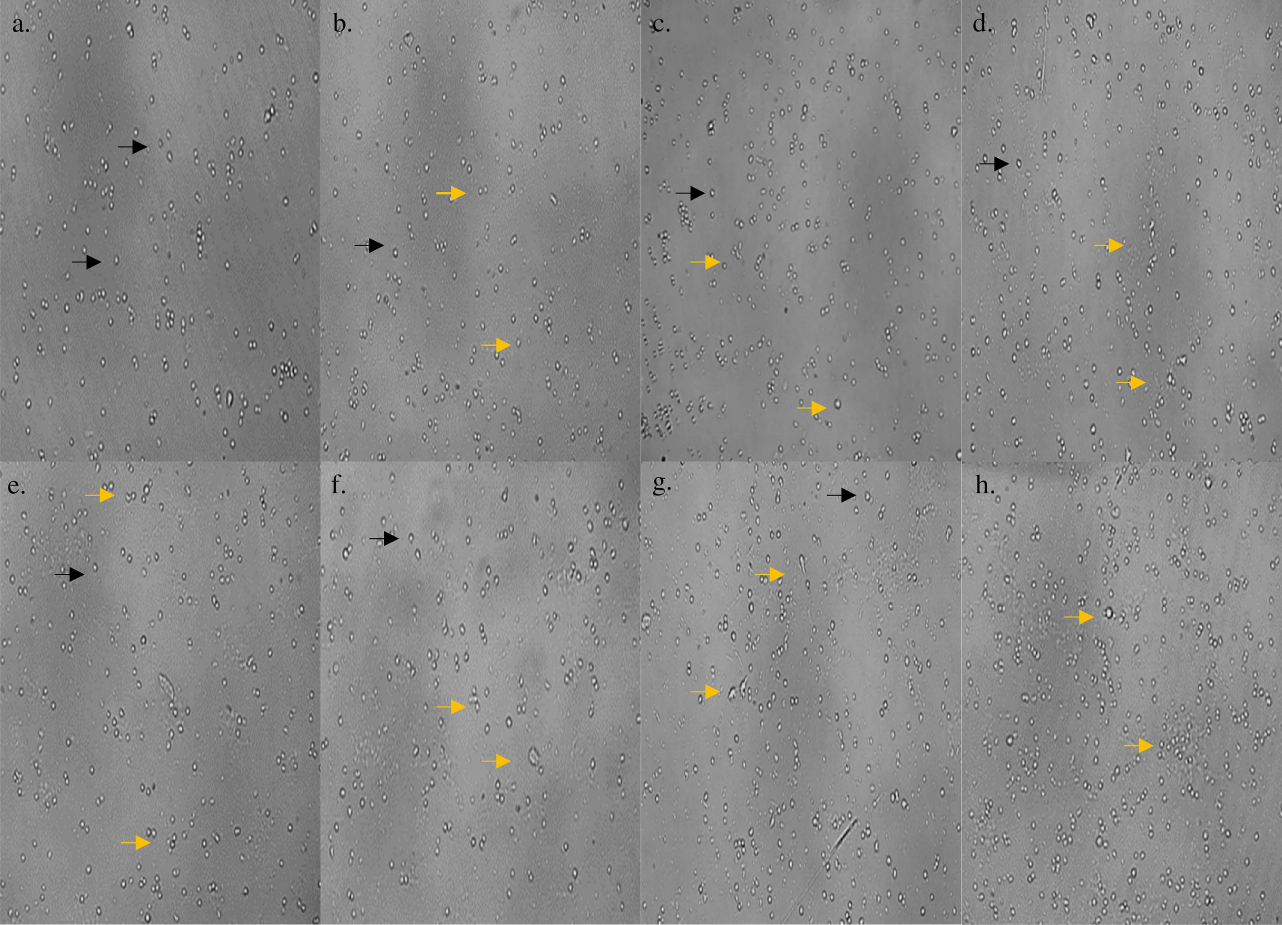
**(a)** Morphology of control THP-1 cells; morphological changes in THP-1 cells on treatment with **(b)** 1 μg/mL; **(c)** 10 μg/mL; **(d)** 50 μg/mL; **(e)** 100 μg/mL; **(f)** 250 μg/mL; **(g)** 500 μg/mL; **(h)** 1,000 μg/mL concentration of *S. epidermidis* SAS1 bioactive metabolites. Normal live cells are indicated by black arrowheads and dead cells and apoptotic cells with indicated by orange arrowheads.

On subjecting the THP-1 cell line to different concentrations (0–1,000 μg/mL) of bioactive metabolites extracted from *S. epidermidis* SAS1, an overall reduction in the viable cells was seen. The lowest number of viable cells, i.e., 13.08 ± 3.49%, were observed at 1000 μg/mL of SAS1 metabolites (Figure 1a). This decrease was significantly lower. An IC_50_ value of 47.52 ± 0.18 μg/mL was observed using the linear regression. Furthermore, cell shrinkage and apoptotic damage was seen in THP-1 cells on analyzing the characteristic morphological changes. Here, the increase in SAS1 metabolites concentration was accompanied by a corresponding rise in the expulsion of cellular contents (Figure 2).

#### 3.3.2 ROS estimation with flow cytometry- THP-1

**Figure 3:**
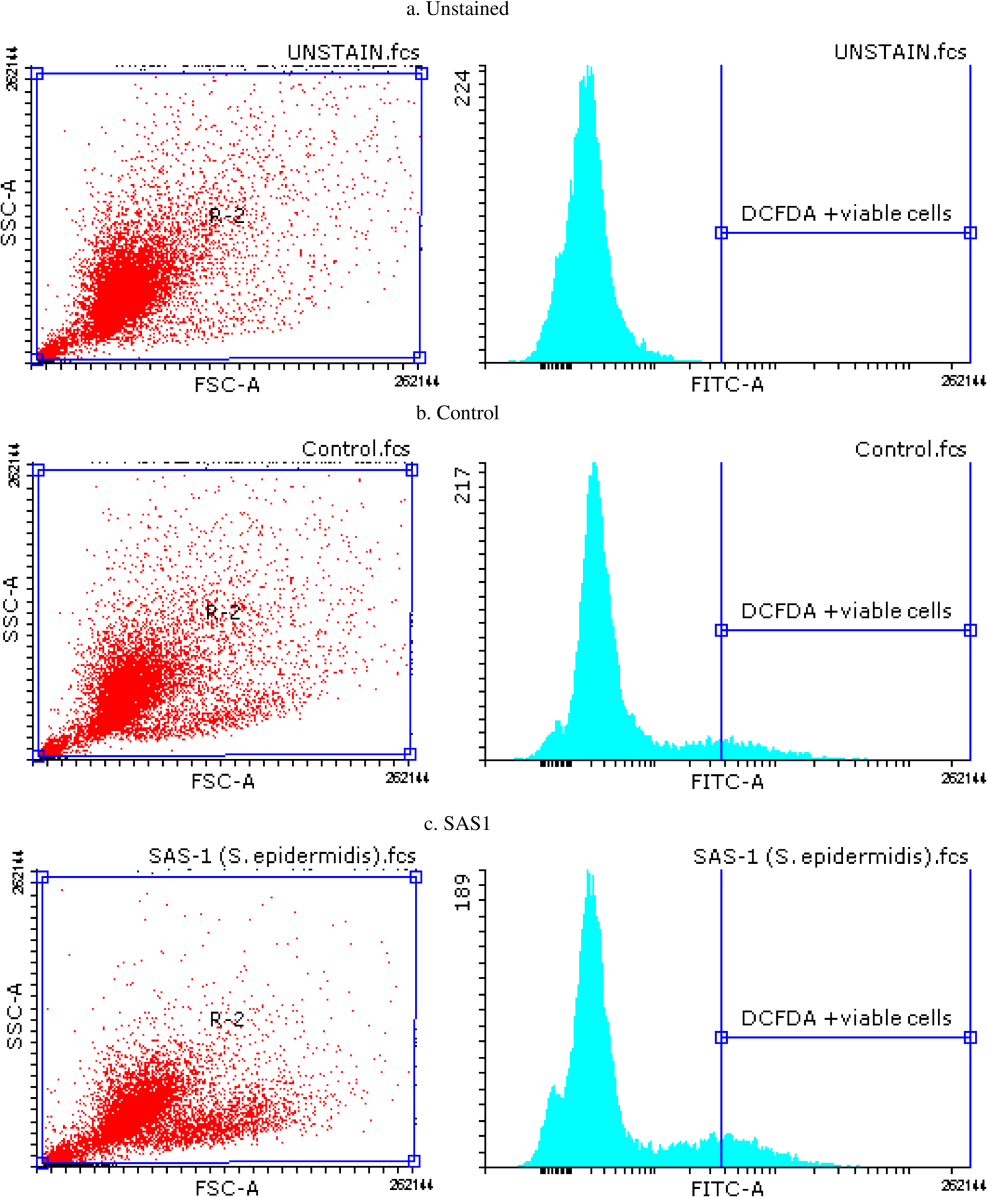
Flow cytometry plots for apoptosis detection in **(a)** Unstained, **(b)** Control and **(c)** *S. epidermidis* SAS1 treated cells.

The ROS Estimation analysis of the THP-1 cell line was done with flow cytometry. The THP-1 cells exposed to *S. epidermidis* SAS1 exhibited a reduction in the number of DCFDA-stained and MFI-positive cells (Figure 1b), which indicates a reduction in the release of reactive oxygen species in comparison. As compared to the control, a 24% reduction in % DCFDA stained cells and a 14% reduction in MFI positive cells was observed in SAS1 metabolites treated cells (Figures 1c, 3).

#### 3.3.3 Protein expression analysis with ELISA (IL-4, IL-5 and IL-13)

**Figure 4:**
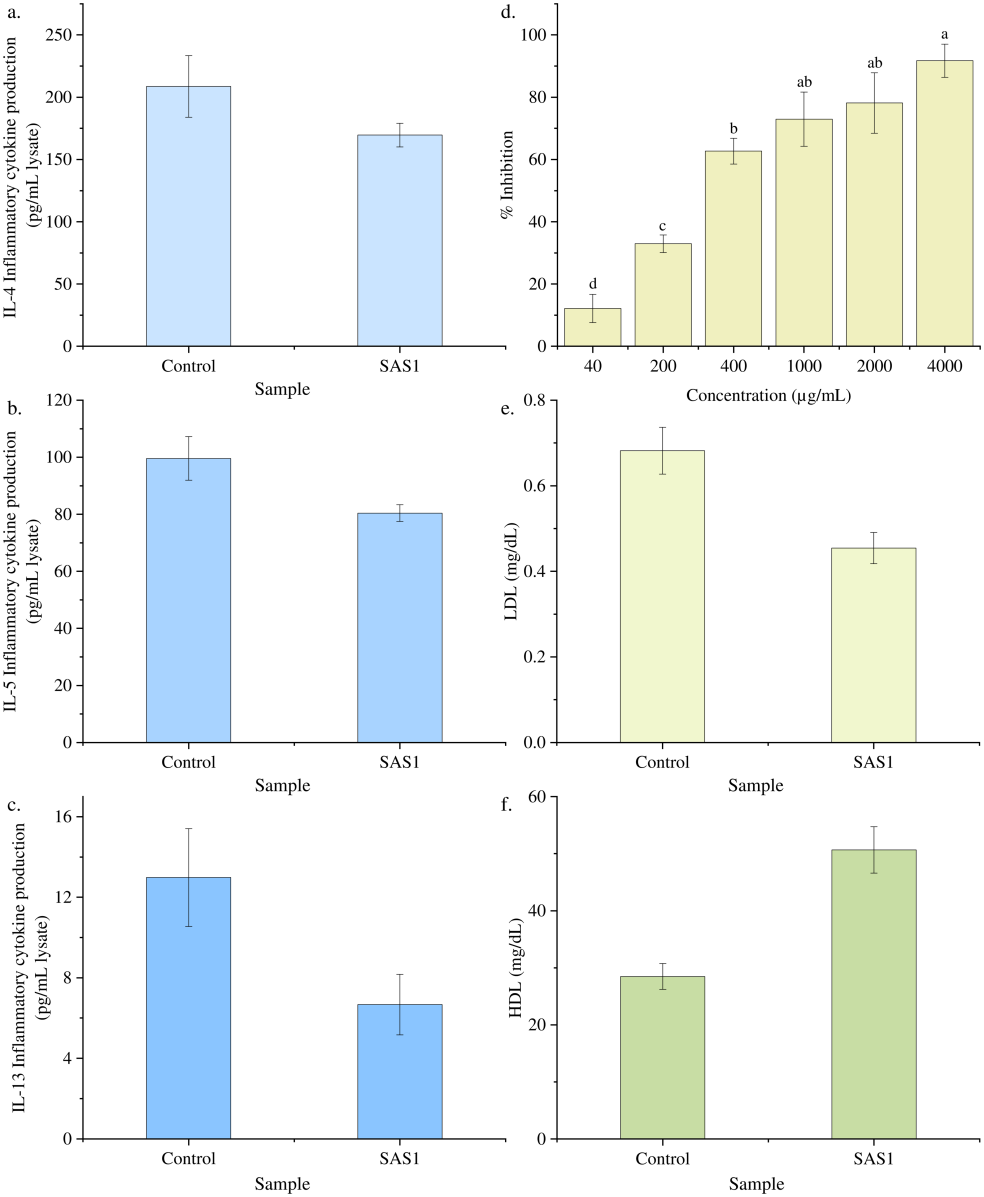
Downregulation of the cytokine at *IC_50_* dose of SAS bioactive metabolites **(a)** IL-4; **(b)** IL-5; **(c)** IL-13; **(d)** Percentage lipase inhibition activity of SAS1 bioactive metabolites. **(e)** Glyceride: LDL cholesterol measurement. **(f)** Glyceride: HDL cholesterol measurement. The data represents mean *n* = 3 ± SD. Mean with different superscript letters are different by Tukey’s *post hoc* test (*p* < 0.05).

It was evaluated in response to varying concentrations (0–2000 pg./mL) of bioactive metabolites extracted from *S. epidermidis*. A downregulation in the production of inflammatory cytokine IL-4 was observed with increasing concentration of bioactive metabolites. Moreover, on treating the cells with the IC_50_ dose of SAS1 metabolites, 169.58 ± 9.42 pg./mL cytokine production was seen, 18% lower than the control’s (Figure 4a). Moreover, the varying concentrations of SAS1 bioactive metabolites were found to be downregulating the production of IL-5. On treating the cells with the IC_50_ dose of SAS1 metabolites, 80.42 ± 2.95 pg./mL cytokine production was observed, 19% lower than the control (Figure 4b). Furthermore, on treating the cells with an IC_50_ dose of SAS1 metabolites, IL-13 inflammatory cytokine production of 6.67 ± 1.50 pg./mL was seen, which was ~48% lower than that of the control (Figure 4c).

### 3.4 Anti-obesity potential of probiotic *Staphylococcus epidermidis* SAS1

#### 3.4.1 In-vitro cytotoxicity evaluation using -3T3 L1 for adipogenesis determination

**Figure 5:**
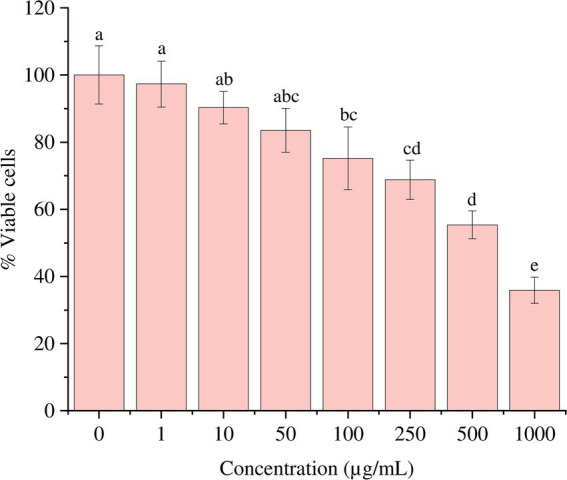
Reduction in the number of viable 3T3 L1 on increasing the exposure of *S. epidermidis* SAS1 bioactive metabolites. The data represents mean *n* = 3 ± SD. Mean with different superscript letters are different by Tukey’s *post hoc* test (*p* < 0.05).

**Figure 6:**
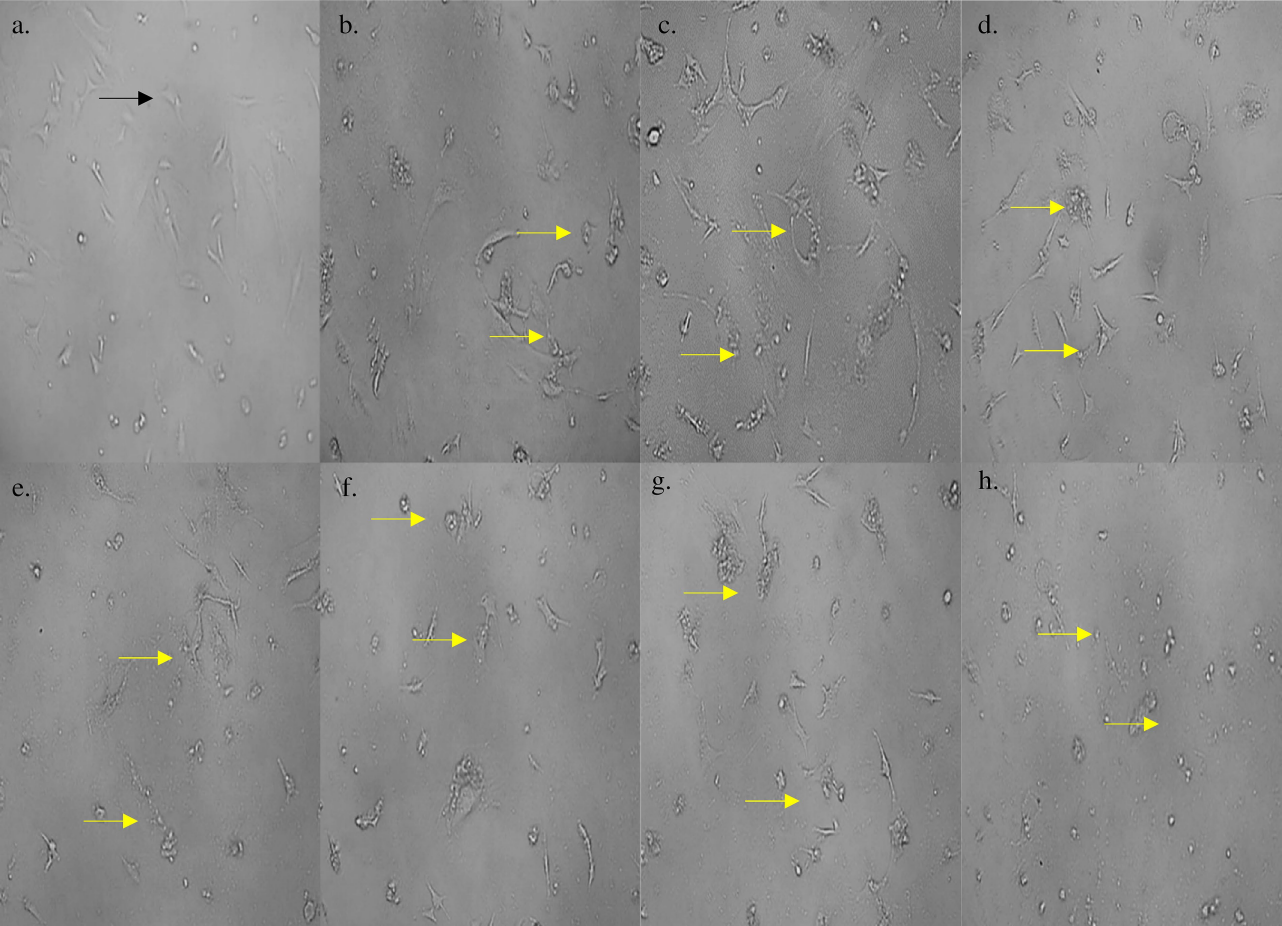
**(a)** Morphology of control 3T3 L1 cells; morphological changes in 3T3 L1 cells on treatment with **(b)** 1 μg/mL; **(c)** 10 μg/mL; **(d)** 50 μg/mL; **(e)** 100 μg/mL; **(f)** 250 μg/mL; **(g)** 500 μg/mL; **(h)** 1,000 μg/mL concentration of *S. epidermidis* SAS1 bioactive metabolites. Normal live cells are indicated by black arrowheads and apoptotic damage and release of their content is indicated by yellow arrowheads.

The cell line 3T3 L1 was subjected to different concentrations (0–1,000 μg/mL) of *S. epidermidis* SAS1 metabolites. The increasing concentration of SAS1 metabolites further reduced the viability of the cells (Figures 5, 6). The lowest number of viable cells, i.e., 35.91 ± 3.85%, was observed at a bioactive metabolite’s concentration of 1,000 μg/mL. An IC_50_ value of 514.4 ± 0.061 μg/mL was observed using the linear regression. The 3T3 L1 cells showed characteristic morphological changes, including cell shrinkage and release of cytoplasmic content with a rise in the concentration of *S. epidermidis* SAS1 bioactive metabolites (Figures 5, 6).

#### 3.4.2 Lipase enzyme inhibition assay

The lipase inhibition activity was estimated, and a linear regression was used to calculate the IC_50_ value. The SAS1 metabolites exhibited an IC_50_ value of 332.6 ± 0.065 μg/mL. 332.6 μg of SAS1 metabolites was found to be equivalent to 3.024 μg of Orlistat (Figure 4d).

#### 3.4.3 Glyceride: HDL and LDL cholesterol measurement

Furthermore, 50.65 mg/dL of HDL cholesterol was observed in SAS1 bioactive metabolites, which was 43% higher than the control. Meanwhile, 0.45 mg/dL LDL cholesterol was seen in SAS1 bioactive metabolites compared to the control, which exhibited 0.68 mg/dL LDL cholesterol (Figures 4e,f).

#### 3.4.4 Triglyceride content

**Figure 7:**
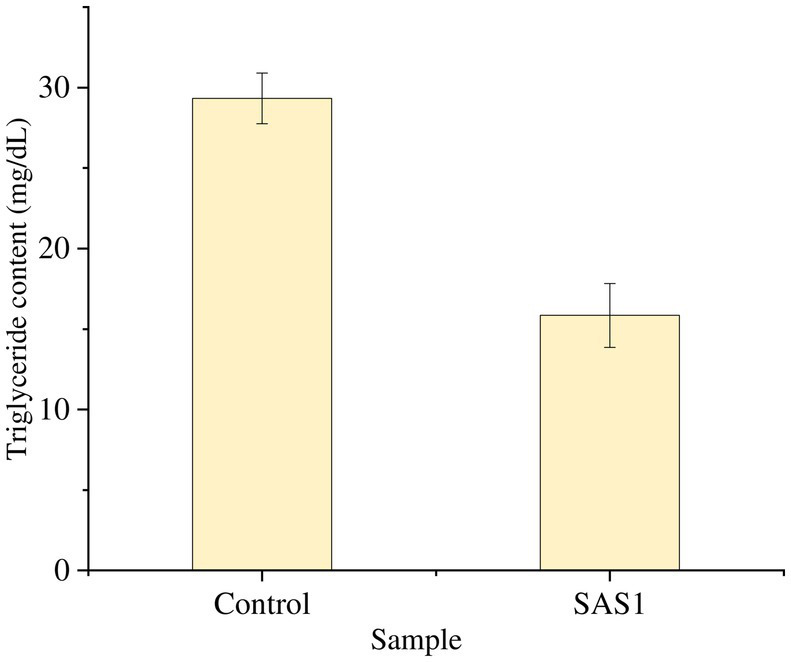
Measurement of triglyceride content. The data represents mean *n* = 3 ± SD.

The triglyceride content in SAS1 bioactive metabolites was 15.84 mg/dL, 45% lower than that of the control (Figure 7).

## 4 Discussion

The present study evaluates the anti-allergic and anti-obesity characteristics of the skin commensal *S. epidermidis* for the first time. Using LC–MS/MS analysis, 75 bioactive metabolites were identified. These included several compounds that were found to have anti-allergic, anti-cancer, and anti-obesity properties, such as (2R,2’R)-2,2′,3,3’-Tetrahydroxy-beta,beta-caroten-4-one (Wu et al., 2023), beta-phellandrene (Thangaleela et al., 2022), Lipoic acid (Andrea Moura et al., 2015; Koh et al., 2011), Norvaline (Zhu et al., 2022; Li et al., 2025), 5-Methoxy-6-methyl-1H-benzimidazole (Lee J. Y. et al., 2023). Additionally, these metabolites have been previously reported for their antioxidant properties, which may contribute to the overall therapeutic potential of *S. epidermidis* SAS1. Techniques like HPLC and mass spectroscopy, can be applied to precisely identify the particular molecule. In the context of allergic diseases, the activation of Th2 (T helper 2) cells, along with CD4 + T lymphocytes and B lymphocytes, leads to the manifestation of symptoms such as eczema, allergic inflammation, nasal itching, sneezing, and congestion (Hoyte and Nelson, 2018). The observed phenomena can be attributed to the significant infiltration of eosinophils and mast cells and the release of bioactive substances from the cells. Increased secretion of various leukocytes, including IL-4, IL-5, and IL-13, as well as specific IgE antibodies (Huang et al., 2022).

An anti-allergic drugs are required to solve the problem by inhibiting the growth of mast cells and eosinophils or reducing the synthesis of bioactive substances and interleukins. The in-vitro cytotoxicity of *S. epidermidis* against the THP-1 cell line was evaluated using an MTT assay. The THP-1 cell line, derived from human leukemia monocytic cells, is widely used to study the regulation of macrophage and monocyte functions (Chanput et al., 2014). The increased concentration of *S. epidermidis* SAS1 bioactive metabolites causes a decrease in both the quantity and altered morphology of THP-1 cells. Furthermore, *S. epidermidis* SAS1 also reduce the secretion of IL-4, IL-5, and IL-13. Watts et al. (2021) investigated the effects of oral administered probiotic *Lactobacilli* on asthmatic rats, and found a decrease in IL-4, IL-5, and eosinophils levels. These findings support the potential role of *S. epidermidis* in modulating immune responses associated with allergic conditions. However, the molecular mechanisms underlying this immunomodulatory effect require further investigation through transcriptomic and proteomic studies.

In a related study, *Lactobacillus gasseri* MG4247 and *Lacticaseibacillus paracasei* strains MG4272 and MG4577 exhibited anti-allergic properties by reducing the concentrations of IL-4, IL-5, and IL-13 in RBL-2H3 mast cells (Lee Y. T. et al., 2023). The effect of reactive oxygen species production was evaluated by flow cytometry, with the medium fluorescence intensity (MFI) was measured. The highest MFI was observed in the amount of ROS after the application of bioactive metabolites to the cell line. ROS includes hydroxyl radicals (OH), superoxide anions (O^2−^), hydrogen peroxide (H_2_O_2_), and singlet oxygen, which are produced during processes such as immunoregulation, toxicity, leukocyte regulation and apoptosis (Nanjundaiah et al., 2016). Notably, the role of ROS in modulating immune responses and adipocyte differentiation remains an important area for future exploration.

Reactive oxygen species (ROS) and reactive nitrogen species (RNS) were detected through flow cytometry after exposing macrophages to probiotic preparations of *Escherichia coli* Nissle 1917 and *Bifidobacterium animalis* subsp. *lactis*, as reported by González-Magallanes et al. (2023). Similarly, Rocha-Ramírez et al. (2017) documented the activation of macrophages to produce ROS through the use of *Lactobacillus helveticus* IMAU70129, *Lacticaseibacillus rhamnosus* GG and KLDS, as well as *L. casei* IMAU60214. Consequently, *Staphylococcus epidermidis* may be a promising candidate for anti-allergic applications.

It has also been evaluated for its potential to act as an anti-obesity agent. There is a strong relationship between excess body fat, cholesterol levels, and obesity. Therefore, strategies to mitigate obesity involves delaying or limiting the digestion and absorption of fats and carbohydrates from dietary sources (Park et al., 2023). This can be done by inhibiting the enzymes responsible for the fats and carbohydrate metabolism (Zhang et al., 2020). In the United States, orlistat, liraglutide, naltrexone/bupropion, and semaglutide are approved for the anti-obesity treatments. Notably, orlistat used as a control in this study, works by inhibiting lipase activity, although it may have effects on the liver and gastrointestinal system (Karale et al., 2022; Müller et al., 2022).

Due to concern regarding the safety and efficacy of pharmaceutical drugs, researchers are looking for natural anti-obesity medications that will provide long-term results. More importantly, probiotics derived from *Lactobacillus* and *Bifidobacterium* strains have been shown to have anti-obesity effects (Kober et al., 2024). Different strains of *Bifidobacterium* species FS31-12, L66-5, M13-4, and L75-4 was examined and reported to lower the serum triglycerides with weight-loss properties (Yin et al., 2010). Similarly, visceral fat lowering effect in mouse model was observed using *B. adolescentis* supplementation (Chen et al., 2012). Song et al. (2023) examined the anti-obesity effect of *Lacticaseibacillus paracasei* AO356 in mice along with the gut biome modulative effects. That lead to the abundance of Bacteroides, *Lactobacillus*, and *Oscillospira* population, might be help in maintaining the gut dysbiosis. However, the gut microbiome alterations associated with *S. epidermidis* supplementation remain unexplored. Future studies should analyze gut microbial shifts and their correlation with metabolic improvements. The in-vitro cytotoxicity potential of *S. epidermidis* against the 3T3 L1 cell line (used to measure anti-adipogenic activity) was evaluated using the MTT assay. The 3T3 L1 cell line represents a murine preadipocyte model for preadipocytic cell differentiation studies (Cave and Crowther, 2019).

Morphological changes and a rise in impaired adipocytes were observed as *S. epidermidis* SAS1 concentration increases. Similar data of increased lipolysis and inhibition of adipogenesis traits were observed when the 3T3 L1 cell line was treated with *Lactobacillus rhamnosus* (Kang et al., 2023). Additionally, the activity of the lipase-inhibiting enzyme was determined. The lipase inhibition enzyme assay using p-nitrophenyl esters as the substrate is the most common and widely used method (Vo et al., 2022).

As the bioactive metabolites concentration increases, lipase inhibition enzyme activity increases. The probiotic strain *Lactiplantibacillus plantarum* MGEL20154 was selected for its potential anti-obesity role due to its ability to inhibit lipase and *α*-amylase activities (Spínola and Castilho, 2021). Similar results were also reported by Kim et al. (2022) investigated *Lactiplantibacillus* plantarum KC as a potential anti-obesity agent. In addition, the glycerol concentration in the culture medium was evaluated to determine the extent of lipolysis. The findings show that *S. epidermidis* decreased LDL levels in 3T3-L1 adipocytes, increased HDL, and maintained good triglyceride levels in 3T3-L1 adipocytes. Comparable results were noted in the investigation of the anti-obesity effects of *Lactobacillus fermentum* CQPC05 in mice fed a high-fat diet (Zhu et al., 2019).

This strain may be a candidate for anti-allergic and anti-obesity probiotic. Further studies are required to carry out the *in vivo* studies to validate the therapeutic potential of SAS1 in real biological systems. Although probiotics are still developing, it is important to regularly assess their safety to ensure that the expected safety profiles align with the needs of individuals who are allergic or overweight. While our findings suggest a low virulence potential of *S. epidermidis* SAS1, additional studies, including biofilm formation assays and in vivo infection models, are needed to confirm its safety and therapeutic applicability. Additionally, our study does not include a direct comparison with other *S. epidermidis* strains, which is a limitation that should be addressed in future research to better contextualize our findings.

## 5 Conclusion

The current study evaluated the *S. epidermidis* SAS1 strain for its potential therapeutic applications as a functional agent possessing anti-allergic and anti-obesity properties. The extract of *S. epidermidis* SAS1 demonstrated a reduction in allergen-induced inflammation and the release of cytokines. It also exhibits cytotoxic effects on THP-1 cells and is often used to study macrophage and monocyte functions. Although probiotics cannot eliminate allergies, they can reduce the frequency and intensity of allergic symptoms. The *S. epidermidis* SAS1 extract also showed significant lipase-inhibiting enzyme activity, suggesting that improving lipolysis and reducing adipogenesis may help obese individuals improve lipid metabolism. Therefore, these findings suggest that such bacteria have the potential to prevent or treat allergies and obesity.

## Data Availability

The datasets presented in this study can be found in online repositories. The names of the repository/repositories and accession number(s) can be found in the article/Supplementary material.
