# Supplementary material for: Staphylococcus epidermidis SAS1: new probiotic candidate for obesity and allergy treatment their mechanistic insights and cytotoxicity evaluation
Source: Front Microbiol. 2025 Apr 30;16:1546687. doi: 10.3389/fmicb.2025.1546687 (PMC12075200; doi:10.3389/fmicb.2025.1546687)
Supplement: Supplementary file 1 [file Table_1.docx]

**
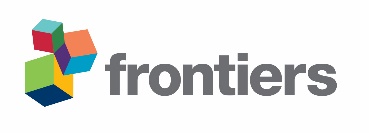
**

Supplementary Material

**Table S1: Biochemical analysis of strain SAS1 (*S. epidermidis*)**

| **S.NO** | **Biochemical tests** | **Observation** |
| --- | --- | --- |
|  | Hemolysis test | Negative |
|  | Urease test | Negative |
|  | Gelatinase test | Negative |
|  | DNase test | Negative |
|  | Mobility test | Negative |
|  | Gram staining | Gram Positive |
|  | Simmons citrate utilisation | Negative |
|  | Triple sugar iron | Negative |
|  | Methyl red | Negative |
|  | Indole | Negative |
|  | VP (Voges–Proskauer) | Negative |
|  | Fructose hydrolysis | Positive |
|  | Lactose hydrolysis | Positive |
|  | Sucrose hydrolysis | Positive |
|  | Catalase test | Positive |
|  | NaCl (10%) | Negative |
