# Supplementary material for: Staphylococcus epidermidis SAS1: new probiotic candidate for obesity and allergy treatment their mechanistic insights and cytotoxicity evaluation
Source: Front Microbiol. 2025 Apr 30;16:1546687. doi: 10.3389/fmicb.2025.1546687 (PMC12075200; doi:10.3389/fmicb.2025.1546687)
Supplement: Supplementary file 2 [file Table_2.docx]

**
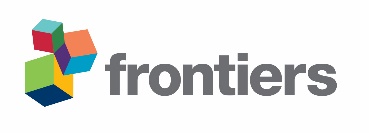
**

Supplementary Material

**Table S2: Identification of secondary metabolites from bacteriocin using LC-MS/MS analysis**

| **Sl. No.** | **Description** | **RT**  **(min)** | **Fragmentation** | **Formula** | ***m/z*** | **Structure** |
| --- | --- | --- | --- | --- | --- | --- |
|  | 1-oleoyl-2-palmitoyl-sn-glycerol | 86.31793333 | 6.25 | C_37_H_70_O_5_ | 594.5037 |  |
|  | 6α-Hydroxytestosterone (MFCD00198760) | 2.688933333 | 7.43 | C_24_H_40_O_11_ | 884.3898 |  |
|  | 16β-hydroxytestosterone | 2.688933333 | 7.96 | C_21_H_24_O_8_ | 300.1480 |  |
|  | MFCD00273002  (Cholesterol β-D-glucoside) | 80.4608 | 4.43 | C_13_H_22_O_2_ | 244.1217 |  |
|  | (3beta)-Cholest-5-en-3-yl alpha-D-glucopyranoside | 80.4608 | 4.43 | C_21_H_22_O_6_ | 244.1599 |  |
|  | 1-Hydroxy-6,8-dimethoxy-3-(2-oxopropyl)-2-naphthaldehyde | 22.22216667 | 2.81 | C_20_H_30_O_5_ | 304.2025 |  |
|  | QS2980000 (3-Pyridinecarboxaldehyde) | 40.89273333 | 10.5 | C_18_H_39_NO_2_ | 974.8762 |  |
|  | Undecanol | 87.834 | 14.1 | C_18_H_39_NO_3_ | 548.3698 |  |
|  | AAF  (2-Acetylaminofluorene) | 37.48223333 | 17.7 | C15H13NO | 223.0997193 |  |
|  | 3-O-(alpha-L-olivosyl)oleandolide | 79.03178333 | 4.09 | C_26_H_44_O_10_ | 516.2922823 |  |
|  | Cyclo(L-Trp L-Pro) | 32.95275 | 7.51 | C_16_H_17_N_3_O_2_ | 283.1306677 |  |
|  | Butyrin | 30.8 | 1.85 | C_15_H_26_O_6_ | 302.1737 |  |
|  | 9α-Lumisterol | 71.15923333 | 0 | C_28_H_44_O | 361.3254 |  |
|  | S-[(1Z)-N-Hydroxy-6-(methylsulfanyl)hexanimidoyl]cysteine | 63.83908333 | 29.1 | C_10_H_20_N2O_3_S_2_ | 392.1532 |  |
|  | L-alpha-Glycerylphosphorylcholine | 63.49523333 | 3.26 | C_8_H_20_NO_6_P | 222.0878 |  |
|  | N-Methylcytidine | 32.21145 | 7.38 | C_10_H_15_N_3_O_5_ | 222.0878 |  |
|  | (5Z)-5-[(1,6-Dihydroxy-2,4-cyclohexadien-1-yl)imino]-5-hydroxy-L-norvaline | 33.22898 | 4.43 | C_11_H_16_N_2_O_5_ | 251.1802 |  |
|  | Anthramycin | 62.5989 | 4.02 | C_16_H_17_N_3_O_4_ | 316.13 |  |
|  | 3,7-Dimethyl-1,6-octadien-3-yl 6-O-beta-D-xylopyranosyl-beta-D-glucopyranoside | 74.81217 | 2.11 | C_21_H_36_O_10_ | 487.1922 |  |
|  | (6R)-5-Amino-3,5-dideoxy-6-[(1S,2S)-1,2,3-trihydroxypropyl]-alpha-L-threo-hex-2-ulopyranosonic acid | 32.71033333 | 2.46 | C_9_H_1_*_7_*NO_8_ | 290.0843 |  |
|  | (1Z,5Z,9E)-12-Isopropylidene-1,5,9-trimethyl-1,5,9-cyclotetradecatriene | 61.66877 | 1.04 | C_11_H_22_N_2_O_8_ | 311.2146 |  |
|  | Mannopine | 32.05643333 | 1.04 | C_11_H_22_N_2_O_8_ | 311.1437 |  |
|  | (5xi,8alpha,9xi,10alpha,13alpha)-Kaur-15-ene | 61.66877 | 0 | C_20_H_32_ | 311.2146 |  |
|  | 9beta-pimara-7,15-diene | 61.66877 | 0 | C20H32 | 311.2146 |  |
|  | 9-Hydroxy-10-methoxy-3,12-didehydrogalanthan-1-one | 65.6994 | 41.7 | C_16_H_17_NO_3_ | 236.1058 |  |
|  | N-Demethylnarwedine | 65.6994 | 41.7 | C_16_H_17_NO_3_ | 236.1058 |  |
|  | (4aS,10bR)-noroxomaritidine | 65.6994 | 41.7 | C16H17NO3 | 236.1058 |  |
|  | Pentadecanal | 72.3318 | 0 | C15H30O | 268.2627 |  |
|  | 2-Pentadecanone | 72.3318 | 0 | C_15_H_30_O | 268.2627 |  |
|  | 9H-Fluoren-9-one | 37.6 | 2.05 | C_13_H_8_O | 222.0914 |  |
|  | L-(+)-Penicillamine | 35.2781 | 23.7 | C_5_H_11_NO_2_S | 191.0846 |  |
|  | MFCD03938942 | 35.2781 | 23.7 | C_5_H_11_NO_2_S | 191.0846 |  |
|  | 1-(beta-D-Ribofuranosyl)-1,4-dihydronicotinamide | 35.39932 | 32 | C_11_H_16_N2O_5_ | 279.0946 |  |
|  | L-Methionine Sulfoximine | 35.70937 | 42.7 | C_5_H_12_N_2_O_3_S | 222.0904 |  |
|  | Cinchonidinone | 35.86438 | 33.5 | C_19_H_20_N_2_O | 334.1905 |  |
|  | S-(1H-Indol-3-ylmethyl)-L-cysteine | 35.96582 | 8.51 | C_12_H_14_N_2_O_2_S | 292.1102 |  |
|  | Cyclo(leucylleucine) | 25.7877 | 5.95 | C_12_H_22_N_2_O_2_ | 265.1304 |  |
|  | Cinchoninone | 27.02788 | 13.8 | C_19_H_20_N_2_O | 293.1637 |  |
|  | N-(3-carboxypropanoyl)-N-hydroxycadaverine | 26.75165 | 3.61 | C_9_H_18_N_2_O_4_ | 183.1122 |  |
|  | (3R,5R,6S,7S,9R,11E,13R,14R)-14-Ethyl-6-hydroxy-3,5,7,9,13-pentamethyloxacyclotetradec-11-ene-2,4,10-trione | 26.4416 | 2.09 | C_20_H_32_O_5_ | 375.2147 |  |
|  | 7,8-Diaminononanoic acid | 26.28658 | 0,957 | C_9_H_20_N_2_O_2_ | 211.1415 |  |
|  | [(2S)-2-{(1R)-1-Hydroxy-2-[(1S,2S,4aS)-3-hydroxy-1,2,4a,5-tetramethyl-4-oxo-1,2,3,4,4a,7,8,8a-octahydro-1-naphthalenyl]ethyl}-2-oxiranyl](oxo)acetaldehyde | 26.04415 | 9.34 | C_20_H_28_O_6_ | 329.1746 |  |
|  | (2R,2'R)-2,2',3,3'-Tetrahydroxy-beta,beta-caroten-4-one | 26.01035 | 25.3 | C_40_H_54_O_5_ | 653.3614 |  |
|  | Prosolanapyrone II | 27.52675 | 14.5 | C_18_H_24_O_4_ | 305.1744 |  |
|  | Ancymidol | 28.26807 | 52.4 | C_15_H_16_N_2_O_2_ | 221.1067 |  |
|  | 3-Nonenal | 28.3695 | 6.51 | C_9_H_16_O | 179.0827 |  |
|  | 7,8-Diaminononanoic acid | 28.49072 | 10.2 | C_9_H_20_N_2_O_2_ | 211.1421 |  |
|  | (5Z)-5-[(1,6-Dihydroxy-2,4-cyclohexadien-1-yl)imino]-5-hydroxy-L-norvaline | 29.04317 | 2.35 | C11H16N2O5 | 279.096 |  |
|  | 12,13-dihydroxyfumitremorgin C | 30.22977 | 5.35 | C_22_H_25_N_3_O_5_ | 376.1659 |  |
|  | Lipoic acid | 36.70712 | 8.28 | C_8_H_14_O_2_S_2_ | 248.078 |  |
|  | β-D-Fructofuranosyl 4-O-(3-methylbutanoyl)-alpha-D-glucopyranoside | 41.91028 | 33.6 | C_17_H_30_O_12_ | 427.1801 |  |
|  | (2S,3R,5S)-3-(2-Aminoethyl)-7-oxo-4-oxa-1-azabicyclo[3.2.0]heptane-2-carboxylic acid | 41.66785 | 0.853 | C_8_H_12_N2O_4_ | 242.1137 |  |
|  | L-Methionine Sulfoximine | 41.29018 | 30.1 | C_5_H_12_N_2_O_3_S | 222.0898 |  |
|  | 5-Methoxy-6-methyl-1H-benzimidazole | 36.98335 | 22.4 | C_9_H_10_N_2_O | 163.0873 |  |
|  | 3,6-Nonadienal | 40.92655 | 29.8 | C_9_H_14_O | 177.0679 |  |
|  | Oxynicotinamide | 43.68315 | 15 | C_6_H_6_N_2_O_2_ | 180.0771 |  |
|  | Adenylthiomethylpentose | 44.14822 | 2.97 | C_11_H_15_N_5_O_3_S | 336.0538 |  |
|  | 7-(3-methylbut-2-enyl)-L-tryptophan | 44.45827 | 43.4 | C_16_H_20_N_2_O_2_ | 295.1414 |  |
|  | 1D-3-amino-1-guanidino-1,3-dideoxy-scyllo-inositol 6-phosphate | 40.36005 | 11.7 | C_7_H_17_N_4_O_7_P | 301.0897 |  |
|  | L-(-)-Camphor | 36.98335 | 65.9 | C_10_H_16_O | 191.0838 |  |
|  | Crinine | 37.25958 | 39.7 | C_16_H_17_NO_3_ | 236.1059 |  |
|  | Cyclopeptine | 37.44842 | 39.7 | C_17_H_16_N_2_O_2_ | 303.1117 |  |
|  | (5E,7E,10R,12Z,14E,17R,18S,22S)-22-Ethyl-10,18-dihydroxy-17-methyloxacyclodocosa-3,5,7,12,14-pentaene-2,16-dione | 37.91348 | 7.44 | C_24_H_34_O_5_ | 441.2027 |  |
|  | beta-D-Fructofuranosyl 4-O-isobutyryl-alpha-D-glucopyranoside | 38.5674 | 4.07 | C_16_H_28_O_12_ | 435.1475 |  |
|  | (-)-beta-phellandrene | 39.03247 | 32.8 | C_10_H_16_ | 175.0889 |  |
|  | Porphobilinogen | 39.15367 | 13.6 | C_10_H_14_N_2_O_4_ | 191.0818 |  |
|  | (2S,3R,5S)-3-(2-Aminoethyl)-7-oxo-4-oxa-1-azabicyclo[3.2.0]heptane-2-carboxylic acid | 39.3087 | 49.2 | C_8_H_12_N_2_O_4_ | 165.066 |  |
|  | 7-(3-methylbut-2-enyl)-L-tryptophan | 25.7877 | 17.2 | C_16_H_20_N_2_O_2_ | 237.1375 |  |
|  | (5Z)-5-[(1,6-Dihydroxy-2,4-cyclohexadien-1-yl)imino]-5-hydroxy-L-norvaline | 3.619067 | 8.55 | C_11_H_16_N_2_O_5_ | 279.0952 |  |
|  | (Z,5Z)-5-{[(1Z,2S)-6-Amino-1-{[(1Z,2R)-1-{[(1R)-1-carboxyethyl]imino}-1-hydroxy-2-propanyl]imino}-1-hydroxy-2-hexanyl]imino}-N-[(2S)-2-amino-1-hydroxypropylidene]-5-hydroxy-L-norvaline | 2.84395 | 17.4 | C_20_H_36_N_6_O_8_ | 511.251 |  |
|  | 2-(6-Amino-9H-purin-9-yl)-alpha-D-psicofuranosyl | 2.601533 | 7.39 | C_11_H_15_N_5_O_5_ | 298.1153 |  |
|  | 2,4-diacetamido-2,4,6-trideoxy-beta-L-altrose | 2.688933 | 15.2 | C_10_H1_8_N_2_O_5_ | 247.13 |  |
|  | Proclavaminic acid | 2.72275 | 21.2 | C_8_H_14_N_2_O_4_ | 244.1296 |  |
|  | (Z)-N-[(2S)-2-{(Z)-[(2S,3S,6R,7S)-2,3-Diamino-1,6,7-trihydroxy-8-{1-[hydroxy(imino)methyl]-2-imino-4-imidazolidinyl}octylidene]amino}-1-hydroxypropylidene]-L-valine | 21.94593 | 7.02 | C_20_H_38_N_8_O_7_ | 525.2756 |  |
|  | (3beta,12beta,16beta,21beta)-16,21,23-Trihydroxy-30-oxo-12,13-epoxyoleanan-3-yl beta-D-glucopyranosyl-(1->2)-[beta-D-glucopyranosyl-(1->4)]-alpha-L-arabinopyranoside | 21.35965 | 19.7 | C_47_H_76_O_20_ | 999.4514 |  |
